# Supratentorial subdural hematoma following microvascular decompression: a report of four cases

**DOI:** 10.1186/s40064-016-2002-2

**Published:** 2016-03-22

**Authors:** Takao Nozaki, Kenji Sugiyama, Tetsuro Sameshima, Hiroshi Kawaji, Hiroki Namba

**Affiliations:** Department of Neurosurgery, Hamamatsu University School of Medicine, 1-20-1 Handayama, Higashi-ku, Hamamatsu, Shizuoka 431-3192 Japan

**Keywords:** Microvascular decompression, Acute subdural hematoma, Remote hemorrhage, Supratentorial hemorrhage

## Abstract

**Introduction:**

Microvascular decompression has become an accepted surgical technique for the treatment of trigeminal neuralgia, hemifacial spasm, and other cranial nerve rhizopathies. However, critical complications still exist, and postoperative hemorrhage is one of the most life threatening complications following microvascular decompression. Most of the hemorrhages occur in the infratentorial region, and we found only four reports of supratentorial acute hemorrhages following microvascular decompression. Here, we report four cases of such hematomas and discuss the potential underlying mechanisms. Moreover, we discuss methods for handling such complications.

**Case description:**

Between 2004 and 2015, four patients developed postoperative hemorrhages, all of which were supratentorial subdural hematomas. The hematomas occurred ipsilaterally in two cases and contralaterally in two cases. All of the patients were treated conservatively and discharged without clinical symptoms.

**Discussion and evaluation:**

Although several intracranial hematomas have been reported distant from the craniotomy site, few reports of remote subdural hematomas after microvascular decompression exist. Draining large amounts of intraoperative cerebrospinal fluid may induce brain shifts and tearing of the small bridging veins. Of our four cases, two were ipsilateral and two were contralateral, and the side of the hemorrhage may suggest possible mechanisms of remote subdural hematomas in microvascular decompression. Although a lateral position for microvascular decompression mainly extends ipsilateral bridging veins, a postoperative supine position can extend bilateral veins equally. Therefore, we assumed that, supratentorial subdural hematomas occurred when the patients were returned to the supine position at the end of the microvascular decompression surgery. We may be able to prevent supratentorial subdural hematomas with the application of sufficient amounts of artificial cerebrospinal fluid immediately after a microvascular decompression.

**Conclusion:**

We suggest that it is important to avoid excessive CSF aspiration and to compensate for the cerebrospinal fluid loss with artificial cerebrospinal fluid adequately in order to avoid subdural hematomas after microvascular decompression. In addition, immediate postoperative CT scan is recommended even if the MVD has performed uneventfully.

## Background

Microvascular decompression (MVD) is a surgical technique used to treat trigeminal neuralgia (TN), hemifacial spasm (HFS), and other cranial nerve rhizopathies. Recent technical advances in MVD have greatly improved patient outcomes. Most of the cranial rhizopathies are not life-threatening, and surgeons should minimize the morbidity and mortality in MVD. However, critical complications still exist, with postoperative hemorrhage being one of the most life-threatening complications following MVD (Hanakita and Kondo 1988; Li et al. 2007). Most of the hemorrhages occur in the infratentorial region (McLaughlin et al. 1999), and we found only four reports of supratentorial acute hemorrhages following MVD (Barker et al. 1996; Hanakita and Kondo 1988; Li et al. 2007; Oh et al. 2008). However, because additional cases may be unreported, the number of cases of supratentorial acute hemorrhages following MVD may be underestimated. At our institute, 156 patients underwent MVD to treat TN and HFS between 2004 and 2015. Four patients developed postoperative hemorrhages, all of which were supratentorial subdural hematomas (SDHs). Here, we describe these four cases and discuss the potential underlying mechanisms and methods for handling such complications.

## Case description

A retrospective review of 156 consecutive cases that underwent MVD for HFS and TN at our institute between January 2004 and September 2015 revealed four patients with postoperative supratentorial acute SDHs.

All of the patients underwent MVDs via the lateral suboccipital infrafloccular approach. A 1-in. retrosigmoid craniotomy was performed. With microscopic visualization, the offending vessels were transposed with fibrin glue and strings of Teflon.

**Fig. 1:**
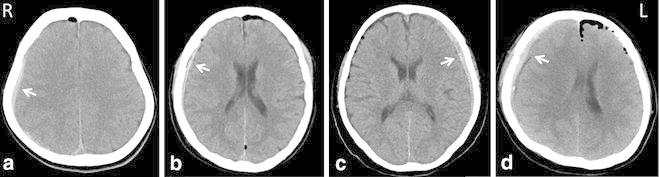
Postoperative computed tomography scans showing unilateral supratentorial subdural hematomas in four patients (*arrows*). **a** Case 1, **b** case 2, **c** case 3, **d** case 4

Four patients had postoperative acute hemorrhages, all of which were supratentorial SDHs. The patients’ clinical characteristics are summarized in Table 1. Preoperative three-dimensional computed tomography was performed in three patients, and magnetic resonance imaging was performed in all four patients. The preoperative imaging and intraoperative observations were normal, except for the compression of the root exit zone of the facial nerve on the affected side by the ipsilateral anterior inferior cerebellar artery. The artery was transposed and attached to the dura mater with fibrin glue and strings of Teflon. The surgical procedures were uneventful, and the patients awoke from anesthesia satisfactorily. None of the patients received antiplatelet or anticoagulant agents, and the patients’ pre- and post-operative blood coagulation parameters were normal. The patients’ perioperative blood pressure levels were kept within the normal range. Postoperative cerebrospinal fluid (CSF) leakage was not observed. Computed tomography that was performed immediately after the operations and/or on the first postoperative day revealed unilateral supratentorial SDHs that were ipsilateral in two patients and contralateral in two patients (Fig. 1a–d). The patients were asymptomatic and treated conservatively. The patients were discharged without any accompanying neural deficits, and they remained asymptomatic during the 6-month follow-up period.

**Table 1:** Summary of four patients with postoperative supratentorial subdural hematoma

Case	Age	Sex	Side of hemifacial spasm	Side of hematoma	Outcome
1	50	Female	Left	Contralateral	No deficits
2	49	Female	Right	Ipsilateral	No deficits
3	74	Female	Right	Contralateral	No deficits
4	51	Male	Left	Ipsilateral	No deficits

## Discussion and evaluation

Here, we describe four cases of supratentorial acute SDHs after MVD that were remote from the site of surgery. Fortunately, the SDH volumes in these cases were relatively small.

Several reports of posterior fossa surgery, including MVD, have noted hemorrhagic complications (Barker et al. 1996; Dubey et al. 2009; Hanakita and Kondo 1988; Kalkanis et al. 2003; Li et al. 2007; McLaughlin et al. 1999; Oh et al. 2008). However, we found only two case reports on the clinical details of supratentorial hemorrhages following MVD (Hanakita and Kondo 1988; Li et al. 2007), and only one was a supratentorial SDH (Hanakita and Kondo 1988). Most large series reports on the hemorrhagic complications of posterior cranial fossa surgery described them insufficiently or with sites in the infratentorial region (Barker et al. 1996; Dubey et al. 2009; Kalkanis et al. 2003; McLaughlin et al. 1999). Two of these reports included one supratentorial SDH (Barker et al. 1996; Oh et al. 2008), but neither described the clinical details of the SDHs.

Several recent cases of postsurgical remote hematomas, including cerebellar hemorrhage after supratentorial craniotomies (Figueiredo et al. 2009; Li et al. 2013) and supratentorial SDH after spinal surgeries (Nowak et al. 2011; Takahashi et al. 2012), have been reported. These reports suggest that the potential risk of remote hemorrhage may be relatively high, even in MVD.

Although the exact causes of postsurgical remote hemorrhages remain unclear, the following factors should be considered: (1) excessive CSF drainage, which may cause brain distortion and laceration of the bridging veins and (2) excessive rotation and flexion of the neck in the lateral decubitus position, which may obstruct jugular venous drainage on the contralateral side and cause subsequent venous hemorrhagic infarction in the supratentorial regions (Hanakita and Kondo 1988; Li et al. 2007).

Interestingly, of our four cases, two were ipsilateral and two were contralateral. The side of the hemorrhage suggests possible mechanisms of remote SDHs in MVD. Although a lateral position mainly extends ipsilateral bridging veins, a postoperative supine position can extend bilateral veins equally. Therefore, we assumed that, when the patients were returned to the supine position at the end of the MVD surgery, low intracranial pressure and/or intracranial air caused the brain to drop, which induced the subsequent supratentorial SDH.

As McLaughlin et al. (1999) indicated, proper exposure and aspiration of CSF from the basal cisterns minimizes cerebellar retraction and are therefore indispensable procedures in MVD surgery. We agree and routinely aspirate sufficient amounts of CSF during MVD. Consequently, supratentorial SDH occurred in four cases. Therefore, as previously suggested (Hanakita and Kondo 1988), excessive CSF drainage should be avoided. We recommend adequate opening of the lateral cerebellomedullary cistern without CSF drainage from cisterna magna. Arachnoid membrane dissection around the jugular foramen to open the cerebellomedullary cistern allows observation of the IXth, Xth, and XIth cranial nerves and the flocculus with unforced cerebellar retraction (Hitotsumatsu et al. 2003). If the flocculus is too large to yield an adequate space, it can be dissected and folded medially (Hitotsumatsu et al. 2003). The neuro-endoscopy can be useful for visualization of REZ and offending vessels, especially in cases with narrow working space (Cheng et al. 2008). In addition, we suggest that, before closure, the craniotomy site should be placed at the highest position as accurately as possible, and the intracranial space should be filled with a sufficient amount of artificial CSF. Since we started to execute this procedure correctly, the postoperative intracranial air in our patients has been remarkably reduced, and no new cases of supratentorial SDHs have occurred. However, only a limited number (20) of cases that were treated with MVD surgery have undergone adequate CSF filling. Thus, additional cases are needed to establish the underlying mechanisms and appropriate procedures in order to prevent supratentorial SDHs. Moreover, even if the surgery was performed uneventfully, immediate postoperative CT scan should be performed routinely.

## Conclusion

Although the serious complications of MVD have been reduced by recent surgical advances, remote SDHs still occur. Excessive CSF aspiration should be avoided, and surgeons should compensate for CSF aspiration with the application of sufficient amounts of artificial CSF immediately after a MVD. In addition, immediate postoperative CT scan is recommended even if the MVD has performed uneventfully.

## Abbreviations

MVD: microvascular decompression
TN: trigeminal neuralgia
HFS: hemifacial spasm
CSF: cerebrospinal fluid
SDH: subdural hematoma
